# Hypoxia awareness and symptom dynamics under aviation-related simulated exposure

**DOI:** 10.3389/fphys.2026.1923338

**Published:** 2026-09-01

**Authors:** Lenka Hanakova, Vladimir Socha, Daniel Hodik, Boris Oniscenko

**Affiliations:** 1 Laboratory of Human Factors and Automation in Aviation, Department of Air Transport, Czech Technical University in Prague, Prague, Czechia; 2 Department of Air Transport Management, Faculty of Aeronautics, Technical University of Kosice, Kosice, Slovakia; 3 Aeromedical Training and Expertise Section, Institute of Aviation Medicine Prague, Prague, Czechia

**Keywords:** aviation, flight, hypoxia, recognition, saturation, subjective evaluation, symptoms

## Abstract

**Introduction:**

Hypoxia poses a significant risk in aviation and other safety-critical domains, yet individual awareness and perception of hypoxic states remain highly variable. This study investigated subjective symptom perception, continuous self-assessment, hypoxia recognition, objective performance, and physiological responses during experimentally induced hypoxia.

**Methods:**

Twenty-three participants completed two experimental sessions in a within-subject design: a control condition and a hypoxia condition. Subjective symptoms were assessed using a post-exposure questionnaire, complemented by continuous ratings of overall feeling, concentration, and breathing. Objective performance was evaluated using a Stroop test and a mental rotation task, while physiological response was quantified via peripheral oxygen saturation (SpO_2_).

**Results:**

Hypoxia induction was robust across all participants, with significant reductions in SpO₂ metrics under hypoxia. While no individual symptoms reached statistical significance after correction for multiple comparisons, several hypoxia-related symptoms showed consistent directional trends and approached statistical significance. Continuous subjective ratings were significantly lower under hypoxia, with breathing demonstrating a pronounced temporal deterioration. Hypoxia recognition occurred more often during hypoxia than during control. No significant effects were observed in objective performance measures.

**Discussion:**

These findings suggest that hypoxia primarily affects physiological state and subjective experience, with breathing-related sensations showing the clearest temporal changes among the subjective measures and potentially contributing to hypoxia awareness within the present experimental paradigm.

## 1 Introduction

Individual recognition of hypoxia is considered a critical factor for flight safety. In military aviation, hypoxia awareness is addressed through recurrent mandatory theoretical and practical training, typically involving hypobaric chambers or reduced oxygen breathing devices (ROBD), with the aim of familiarizing pilots with their individual hypoxic symptoms (North Atlantic Treaty Organization, 2024). In contrast, in civilian aviation, hypoxia education is usually limited to theoretical instruction, with substantial variability across operators and training organizations. Practical hypoxia exposure is rarely included in standard civilian training curricula.

Evidence from military aviation clearly demonstrates the benefits of hypoxia awareness training. Studies consistently report that without prior training, only a minority of pilots are able to reliably recognize hypoxic symptoms, whereas following structured training, recognition rates increase markedly, in some cases exceeding 90% (Leinonen et al., 2020; Chiang et al., 2021b). Moreover, recurrent exposure appears to be essential, as the ability to recognize hypoxia deteriorates over time without reinforcement. Consequently, military aviation authorities typically mandate fixed intervals for hypoxia awareness training, with NATO specifying a maximum interval of five years between training sessions due to the gradual decline over time in pilots’ ability to effectively and promptly recognize their hypoxic state (Leinonen et al., 2020; Woodrow et al., 2011; Artino et al., 2006; Chiang et al., 2021b).

Both hypobaric and normobaric hypoxia induction, as well as hypoxia recognition, have therefore been studied predominantly within the military aviation domain. In this context, hypoxia has been examined not only in terms of physiological consequences but also with respect to cognitive performance, stress responses, and subjective symptom perception. Across studies, hypoxia exposure is generally associated with increased symptom prevalence, elevated stress, and degraded performance (Bouak et al., 2018; Mitchell et al., 2019; Woodrow et al., 2011; Van der Post et al., 2002; Ray et al., 2019; Artino et al., 2006, Artino et al., 2009). However, the magnitude of these effects varies substantially between studies. A key and well-documented feature of hypoxia is the pronounced inter-individual variability in both the onset and manifestation of symptoms, with symptom profiles differing widely between individuals and experimental protocols (Bouak et al., 2018; Mitchell et al., 2019; Woodrow et al., 2011; Ray et al., 2019; Chiang et al., 2021b). Furthermore, the effectiveness of hypoxia awareness training itself appears to depend on factors such as prior experience, occupational role, training background, and ethnic-genetic diversity of the studied populations (Mitchell et al., 2019; Bustamante-Sánchez et al., 2018; Ray et al., 2019).

Despite the extensive military-focused literature, subjective symptom perception and the ability to consciously recognize hypoxia have often been treated as secondary outcomes, typically assessed alongside primary endpoints such as cognitive or motor performance. Large-scale studies frequently rely on retrospective symptom reporting following hypobaric chamber training, providing valuable descriptive data but limited insight into condition-specific differences within individuals (Woodrow et al., 2011). Experimental studies directly comparing subjective perception between hypoxic and non-hypoxic conditions within the same individuals remain relatively limited.

Outside military aviation, the lack of practical hypoxia training and the limited availability of civilianfocused data represent a notable gap. Given that subjective symptom recognition is one of the primary defenses against insidious hypoxia, improved understanding of how individuals perceive and interpret hypoxic symptoms may have important implications for flight safety. This includes potential benefits for crew cross-monitoring, enhanced self-awareness, and the future implementation of practical hypoxia awareness training in civilian aviation.

Against this background, the present study focuses on the subjective perception and recognition of hypoxia under controlled experimental conditions. By combining post-exposure symptom questionnaires, continuous subjective evaluation during exposure, objective cognitive performance measures, and physiological verification of hypoxia via oxygen saturation, the study aims to provide a more detailed characterization of subjective hypoxia recognition and its relationship to physiological and performance-related changes.

## 2 Methods

### 2.1 Participants

The study group consisted of 23 participants (mean age 22.8 ± 2.6 years, body mass 77.5 ± 15.7 kg, height 180.7 ± 12.2 cm). Health and fitness requirements were comparable to those of a Class 1 medical certificate as defined by Commission Regulation (EU) No. 1178/2011, Annex IV (Part-MED). Seventeen participants held a valid Class 1 medical certificate and had been actively piloting within the last 12 months. The sample was selected to reflect an aviation-relevant population with medical fitness characteristics comparable to operational flight personnel. Although not all participants were active professional pilots, the inclusion criteria were designed to ensure relevance to occupational aviation medicine and hypoxiaawareness assessment in fit-to-fly populations. None of the subjects had prior experience with formal hypoxia-recognition training, while 20 subjects reported no or only minimal experience with deliberate hypoxia exposure.

All participants were informed about the principles of the experiment, anonymized data collection, and the potential risks associated with induced hypoxia, in accordance with ethical principles for research involving human subjects (World Medical Association, 2013). The study was approved by the institutional ethics committee, and all participants voluntarily agreed to take part after receiving sufficient information and providing written informed consent. Subjects were instructed to immediately report if hypoxia symptoms became intense enough to warrant interruption of the test. None of the participants reached the predefined stopping criteria during hypoxia exposure. One participant did not complete the study because they declined to participate in the second experimental session for reasons unrelated to safety or hypoxia symptoms and was therefore excluded from the final analyses. Consequently, 24 participants were recruited, of whom 23 completed the study and were included in the final analyses. The experiment was conducted under the supervision of qualified medical personnel.

### 2.2 Experimental procedure

Each participant completed two measurement sessions: one under normal conditions (control, altitude of 230 m) and one under hypoxia. For hypoxia simulation, a reduced oxygen breathing device (ROBD) Hypoxico HYP123 (Hypoxico, Inc., New York, US) was used. Both sessions were conducted using the ROBD to avoid bias introduced by breathing through a mask under different conditions. In the control session, the ROBD was set to produce air without reduced oxygen content. For the hypoxic session, a 9% oxygen mixture was selected, corresponding to an altitude of 20300 ft (6200 m).

Each session lasted 15 minutes, and the two sessions were separated by a minimum 15 minute break. For the testing, fixed order of sessions was applied, with the control session first and the hypoxic session second. This approach was chosen as a new experience with hypoxia could potentially lead to increased subjective awareness and artificially elevate scoring in the control session if it were conducted second. With this order, participants naive to hypoxic exposure first experienced normal conditions and were therefore less likely to “search” for symptoms that were not yet familiar to them. Participants were not informed that the order was fixed and were not told afterwards.Participants were informed only that they would complete two experimental sessions, without being told which session involved hypoxia. In addition, individual participants did not meet during data collection.

**Figure 1:**
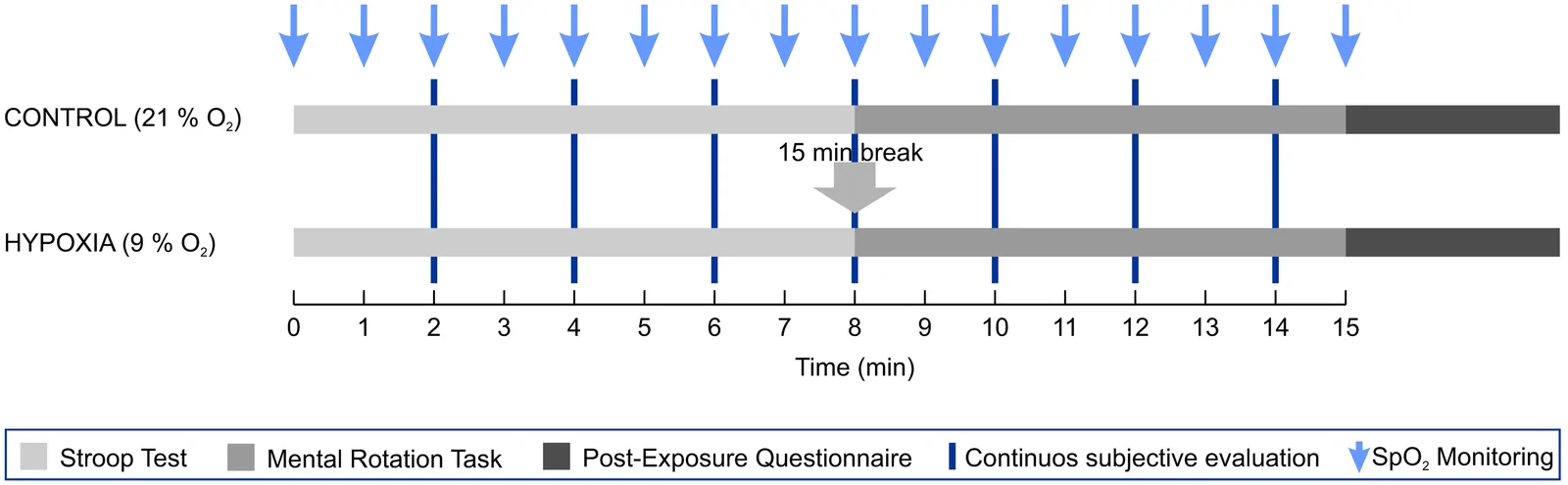
Schematic overview of the experimental schedule for one participant, including control and hypoxia sessions, task sequence, SpO_2_ monitoring and subjective evaluations.

SpO_2_ was recorded once per minute using a fingertip pulse oximeter, as the primary purpose of these measurements was to confirm the hypoxic exposure and ensure participant safety rather than to characterize the detailed temporal dynamics of oxygen desaturation. For these measurements, an SP 55 oximeter was used (SilverCrest, Neckarsulm, Germany). The manufacturer specifies an accuracy of ±2% within the 70–100% SpO_2_ range. The oximeter was attached throughout the experiment, as it is needed for medical monitoring; however, the values were recorded once per minute. The frequency may appear low, but SpO_2_ is used primary for the safety monitoring, and it does not serve as the primary endpoint of the study. The entire experiment was conducted at the Institute of Aviation Medicine in Prague and was supervised by a medical doctor specializing in aviation physiology. Physiological parameters were evaluated in real time by the medical supervisor and recorded at predefined time points. A graphical timescale of a single subject is presented in **Figure 1**.

The primary objective of the study was the subjective recognition of hypoxic symptoms. To add more variability to the symptom spectrum, two tasks were introduced to increase mental workload of subjects. These tasks served primarily as a tool for participants to increase participants’ cognitive workload and to recognize that they may be affected by hypoxia. Therefore, two cognitive tasks were used, administered by proprietary software running on a laptop. The time needed to answer (reaction time) and the accuracy of the answer were recorded.

The first test, the Stroop test (ST) (Stroop, 1935), is widely used for measuring attention and executive functions. Words naming different colours are displayed in a conflicting colour, e.g., the word “green” is displayed in blue. Participants must select the correct colour of the text, not the meaning of the word. Five colours were used: red, green, blue, yellow, and pink. The task requires participants to inhibit automatic responses, thereby engaging executive control and active processing of the sensory stimulus.

As the second test, a mental rotation (MR) task was used. It was a modified version of a broader test battery (PsyToolkit) (Collins and Kimura, 1997). A graphic shape is presented to the participant along with two other shapes. One is rotated around the center of the original shape in the 2-D plane. The other is mirrored and cannot be rotated to exactly “cover” the original. The referenced test included various differences between shapes; only mirroring was included. Around 70 different shapes were carefully prepared not to be symmetrical, as symmetry would generate false answers. The rotation should fall within a certain range (Li et al., 2021) (60—300^◦^ in the software implementation used in the present study), as it would be trivial to recognize shapes too close to the original orientation.

The selected cognitive tasks were chosen to assess cognitive domains relevant to pilot performance. In particular, the Stroop task evaluates executive control, selective attention, and response inhibition, whereas the mental rotation task assesses visuospatial processing. These functions are closely related to the competencies emphasized in the current EASA Knowledge, Skills and Attitudes (KSA) framework (European Union Aviation Safety Agency (EASA), 2020), including problem solving and decision making, situation awareness, and workload management. Although these laboratory tasks do not capture the full complexity of piloting, they assess cognitive processes that are directly relevant to aviation performance.

Because both tasks were time-limited rather than trial-limited, the number of completed trials varied across participants. In the ST, participants completed 179.1 ± 33.6 trials (range: 119–223) under the control condition and 194.0 ± 34.9 trials (range: 127–257) under hypoxia, corresponding to a paired mean difference of 14.9 ± 19.1 trials (hypoxia minus control). In the mental rotation task, participants completed 64.0 ± 20.3 trials (range: 27–100) under the control condition and 67.6 ± 24.6 trials (range: 27–113) under hypoxia, corresponding to a paired mean difference of 3.6 ± 12.0 trials.

In addition, subjective state was continuously evaluated during the 15-minute testing period. Every 2 minutes (i.e., yielding seven ratings per session), participants rated their subjective state using three simple questions. Furthermore, participants were instructed to explicitly report the time point at which they became aware of experiencing a hypoxic state.

The entire testing procedure was conducted using custom-designed software, which provided a synchronized timeline, administered the Stroop and mental rotation tasks, and collected the ongoing subjective ratings. After completion of the 15-minute experimental session, an additional questionnaire focused on specific hypoxia-related symptoms was administered.

### 2.3 Subjective evaluation

Hypoxia recognition was evaluated using three complementary approaches: repeated real-time subjective ratings during exposure, a post-exposure symptom questionnaire, and the recorded time of first hypoxia awareness.

During each session, participants performed a brief self-assessment every two minutes using three questions, each rated on a 1–10 scale (1 – worst, 10 – best): overall subjective state (“How do you feel?”), perceived concentration (“How would you rate your concentration?”), and breathing comfort (“How comfortable are you with your breathing?”). The questions were administered in Czech; the English translation reflects the original meaning.

Participants were additionally instructed to report the first moment at which they subjectively recognized that they were experiencing hypoxia based on symptoms or perceived performance degradation. The corresponding time was recorded.

After each 15-minute session, a post-exposure questionnaire assessing 15 hypoxia-related symptoms was administered. Several hypoxia symptom checklists have been published in the aerospace medicine literature and demonstrate substantial overlap in the principal subjective manifestations of hypoxia (Sampson and Kobrick, 1980; Kumar et al., 2013; Chiang et al., 2021a). The questionnaire used in the present study was based on the symptom framework proposed by Woodrow et al. (2011); however, conceptually overlapping symptoms were merged to improve linguistic clarity and avoid redundancy in the Czech version of the questionnaire. Each symptom was rated using a four-point intensity scale (0 – not present, 1 – mild, 2 – moderate, 3 – strong). The full symptom list included the following: lightheadedness/dizziness, confusion/disorientation, tingling in the fingers, impaired vision, shortness of breath/air hunger, anxiety, cyanosis, euphoria, fatigue, headache, palpitations, rapid or abnormal breathing, hot and cold flashes, muscle weakness/incoordination, and nausea.

The post-exposure symptom analyses were considered exploratory and hypothesis-generating, aiming to identify symptom patterns associated with hypoxic exposure rather than to establish definitive evidence for individual symptom effects.

### 2.4 Data assessment and processing

Data acquisition and processing were performed using custom-designed software providing synchronized experimental timing. For the post-exposure symptom questionnaire, 15 ratings (0–3) were recorded. To assess symptom occurrence under hypoxia, ratings were dichotomized into absence (0) and presence (≥ 1).

For the real-time subjective assessment, the 3 (3 questions, 7 repetitions of each) values on the 1–10 scale were recorded. The continuous subjective evaluation was then processed into a form, allowing evaluation through the whole experimental session. First, the area under curve (AUC) was calculated by numerical integration of the ratings over time using the trapezoidal rule (Equation 1), as integrated in MATLAB:

(1) $$AUC=\int_{0}^{T}f(t)\;\text{d}t\approx \sum_{i=1}^{N-1}\frac{f(t_{i})+f(t_{i+1})}{2}\;(t_{i+1}-t_{i}),$$

where *f*(*t*) denotes the subjective rating at time *t*, *T* is the total duration of the experimental session, and *N* is the number of rating time points. This metric reflects the cumulative subjective experience across the exposure period and accounts for both the magnitude and temporal evolution of the ratings. Second, the mean of the ratings across exposure was calculated for further evaluation.

Third, temporal dynamics were quantified by computing individual linear slopes of the subjective ratings over time for each participant and experimental condition. For each time series, a simple linear regression model (Equation 2) of the form.

(2) $$f(t)=\beta_{0}+\beta_{1}t$$

was fitted, where *t* represents time, *β*_0_ the intercept corresponding to the baseline rating at the beginning of exposure, and *β*_1_ represents the slope of the ratings over time and serves as an index of temporal change during the exposure.

For the ST and MR tasks, reaction times in seconds were recorded, while binary correctness was also collected. The duration of ST was 8 minutes, the duration of MR was 7 minutes. Thus, each participant in each experimental session could have different number of trials. However, the predefined task duration was considered more important than a fixed number of completed trials.

The data were evaluated using three performance metrics. First, accuracy was expressed as the percentage of correct responses. Second, reaction time was quantified as the mean reaction time of correct responses only (Davidson et al., 2003). This approach was selected to ensure an interpretable measure of processing speed that is not confounded by incorrect responses.

Third, a composite performance metric was computed by correcting the mean reaction time across all responses for accuracy, thereby accounting for potential speed–accuracy trade-offs. Accuracy-corrected reaction time was calculated according to Equation 3 (Townsend and Ashby, 1983; Rach et al., 2011).

(3) $$RT_{\text{corr}}=\frac{RT\cdot N_{\text{total}}}{N_{\text{correct}}},$$

where *RT* denotes the mean reaction time across all responses, *N*_total_ denotes the total number of responses, and *N*_correct_ denotes the number of correct responses for a given participant, task, and experimental condition. This metric penalizes both slower responses and increased error rates.

For physiological data, SpO_2_ values recorded once per minute were analyzed. Median and interquartile range were computed for visualization. For statistical analysis, summary metrics including AUC, mean and linear slope were calculated for each participant and condition. However, the mean SpO_2_ value is not an optimal descriptor under hypoxic exposure, as SpO_2_ typically decreases continuously over time. Therefore, the minimum SpO_2_ value observed during each session was also calculated for each participant to reflect peak desaturation.

### 2.5 Statistical analysis

The primary analyses focused on subjective symptom ratings under control and hypoxia conditions. Ordinal mixed-effects models were initially considered but showed convergence issues due to sparse highseverity categories. Therefore, individual symptoms were analyzed using paired Wilcoxon signed-rank tests. The p-values were adjusted for multiple comparisons using the Benjamini–Hochberg false discovery rate (FDR) procedure (Benjamini and Hochberg, 1995). Effect sizes were expressed as matched-pairs rank-biserial correlations. Ninety-five percent confidence intervals (95% CIs) for rank-biserial correlations were estimated using percentile paired bootstrap resampling with 10000 resamples (Efron, 1979).

Differences in symptom occurrence (presence vs. absence) were analyzed using exact McNemar tests for paired binary data (Siegel, 1956), with FDR correction applied across symptoms. For continuous subjective ratings (AUC, mean, slope), differences between conditions were assessed using paired Wilcoxon signedrank tests with matched-pairs rank-biserial effect sizes, 95% CIs, and FDR correction applied within each metric family. Performance metrics from the Stroop and mental rotation tasks were analyzed using paired Wilcoxon signed-rank tests with rank-biserial effect sizes, 95% CIs, and FDR correction applied separately for each task. For SpO_2_ summary metrics, paired Wilcoxon signed-rank tests with rank-biserial effect sizes, 95% CIs, and FDR correction were applied. Associations between physiological hypoxic load and subjective breathing ratings under hypoxia were examined using Spearman rank correlation. Ninety-five percent confidence intervals for Spearman correlation coefficients were estimated using percentile bootstrap resampling with 10000 resamples (Ruscio, 2008). In the association analyses, different SpO_2_ metrics were related to subjective breathing measures according to their physiological interpretation. While AUC and slope were paired with corresponding cumulative and temporal subjective measures, minimum SpO_2_ was related to mean breathing ratings as an indicator of peak desaturation, which was considered more relevant to perceived respiratory discomfort than average oxygen saturation across the entire session. FDR correction was applied to all correlation analyses.

All statistical analyses were performed in MATLAB 2024a (MathWorks, Natick, MA, USA).

## 3 Results

### 3.1 Post-exposure questionnaire

**Figure 2:**
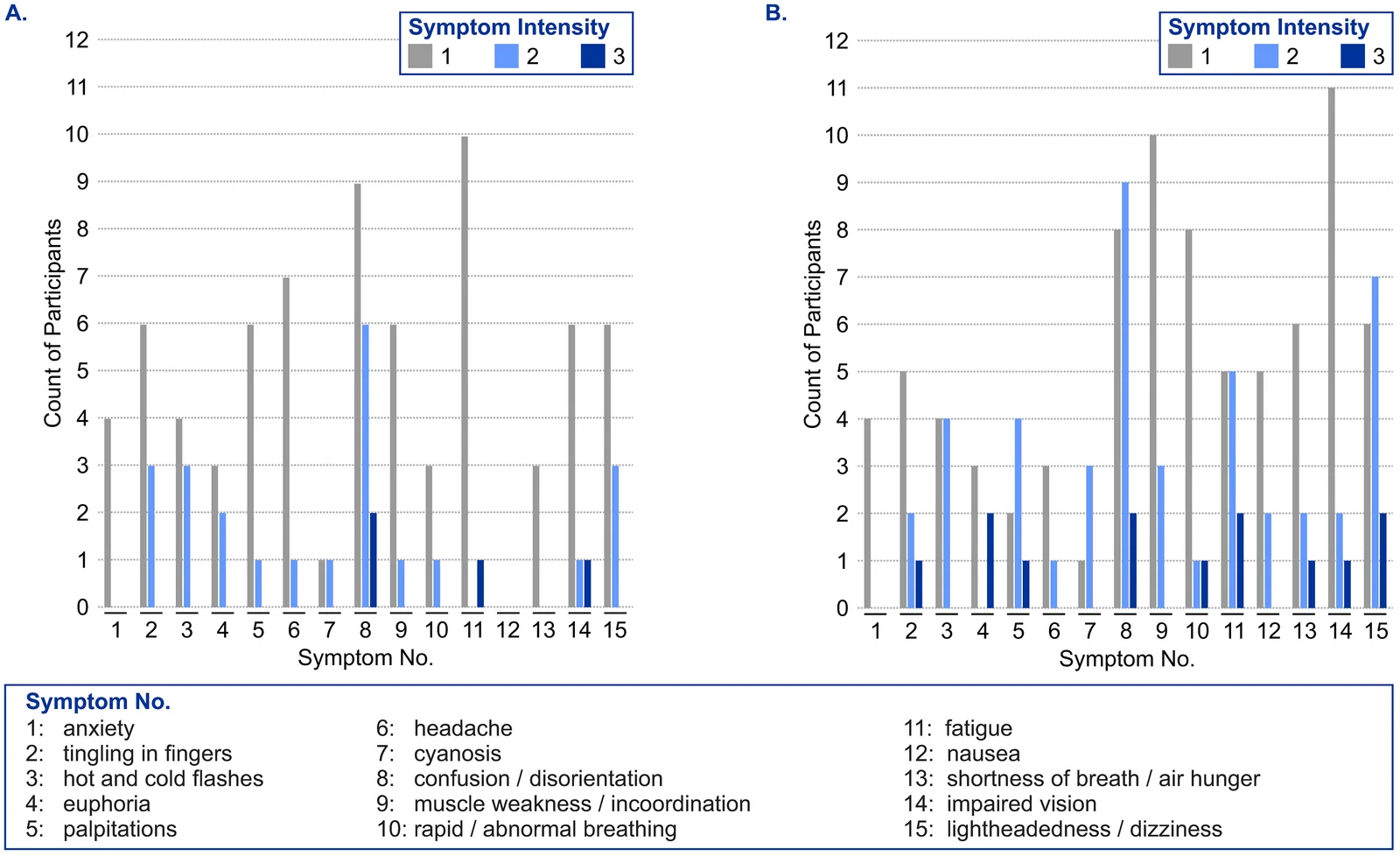
Histograms of reported subjective symptom intensities obtained from the post-exposure questionnaire during the control condition **(A)**, and during the hypoxia condition **(B)**.

Based on the statistical analysis, none of the symptoms reached statistical significance after FDR correction when comparing the control and hypoxia conditions. Nevertheless, several symptoms showed a consistent directional trend towards higher severity under hypoxia (**Table 1**). For impaired vision, shortness of breath/air hunger, and nausea, all non-zero paired differences indicated higher symptom severity under hypoxia, although these results were based on seven non-zero paired differences. Examination of the symptom intensity distributions (**Figure 2**) further supports this pattern, with lightheadedness/dizziness, impaired vision, shortness of breath/air hunger, and nausea tending to be reported more frequently and at higher intensity under hypoxia than during the control condition.

**Table 1:** Comparison of subjective symptoms between control and hypoxia conditions.

Symptom	Non-zero pairs (n)	Median diff.	*r_rb_*	95% CI	*p*_FDR_
Lightheadedness/dizziness	14	1	0.77	[0.38, 1.00]	0.059
Impaired vision	7	0	1.00	[1.00, 1.00]*	0.059
Shortness of breath/air hunger	7	0	1.00	[1.00, 1.00]*	0.059
Nausea	7	0	1.00	[1.00, 1.00]*	0.059
Fatigue	9	0	0.80	[0.33, 1.00]	0.105
Rapid/abnormal breathing	8	0	0.81	[0.29, 1.00]	0.137
Muscle weakness/incoordination	13	0	0.57	[0.08, 1.00]	0.163
Confusion/disorientation	9	0	0.47	[−0.25, 1.00]	0.373
Cyanosis	3	0	1.00	[1.00, 1.00]*	0.373
Headache	6	0	−0.67	[−1.00, 0.00]	0.373
Palpitations	10	0	0.45	[−0.20, 1.00]	0.373
Euphoria	8	0	0.19	[−0.57, 1.00]	0.938
Hot and cold flashes	7	0	0.14	[−0.67, 1.00]	0.938
Tingling in fingers	9	0	0.00	[−0.75, 0.67]	1.000
Anxiety	2	0	0.00	[−1.00, 1.00]	1.000

*All non-zero paired differences were in the same direction, resulting in *r_rb_*= 1.00 and corresponding confidence intervals collapsed to the observed effect size.

*Note.* Median diff. denotes the median of within-subject differences (hypoxia minus control). Non-zero pairs (n) denote the number of participants with a non-zero paired difference and therefore contributing to the estimation of the matched-pairs rank-biserial correlation. *r_rb_*denotes the rank-biserial correlation, with positive values indicating higher symptom severity under hypoxia and negative values indicating lower symptom severity under the control condition. 95% CIs denote percentile bootstrap confidence intervals. *p*_FDR_ indicates FDR-adjusted *p*-values.

Symptoms are ordered by increasing FDR-adjusted p-values. Comparisons were performed using paired Wilcoxon signed-rank tests with false discovery rate (FDR) correction.

To complement the analysis of symptom severity, symptom prevalence was examined by assessing whether a symptom was reported at least once during a given session. As shown in **Table 2**, several symptoms, including impaired vision, shortness of breath/air hunger, rapid or abnormal breathing, nausea, and lightheadedness/dizziness, were reported by a higher proportion of participants under hypoxia compared to the control condition. The largest absolute increase in prevalence was observed for nausea, which was not reported in the control condition but was reported by approximately 30% of participants under hypoxia.

**Table 2:** Prevalence of subjective symptoms in control and hypoxia conditions.

Symptom	Control (%)	Hypoxia (%)	ΔPrevalence (%)	*p*_FDR_
Impaired vision	34.8	60.9	+26.1	0.117
Shortness of breath/air hunger	13.0	39.1	+26.1	0.117
Rapid/abnormal breathing	17.4	43.5	+26.1	0.117
Nausea	0.0	30.4	+30.4	0.117
Lightheadedness/dizziness	39.1	65.2	+26.1	0.211
Muscle weakness/incoordination	30.4	56.5	+26.1	0.365
Headache	34.8	17.4	−17.4	0.469
Euphoria	8.7	17.4	+8.7	0.938
Confusion/disorientation	73.9	82.6	+8.7	1.000
Tingling in fingers	39.1	34.8	−4.3	1.000
Anxiety	17.4	17.4	0.0	1.000
Cyanosis	21.7	21.7	0.0	1.000
Fatigue	47.8	52.2	+4.3	1.000
Palpitations	30.4	30.4	0.0	1.000
Hot and cold flashes	30.4	34.8	+4.3	1.000

Symptom occurrence was defined as a rating ≥ 1. Differences were analyzed using the exact McNemar test with false discovery rate (FDR) correction. Symptoms are ordered by increasing FDR-adjusted p-values.

However, none of the prevalence differences remained statistically significant after correction for multiple comparisons using the false discovery rate (FDR).

**Figure 3:**
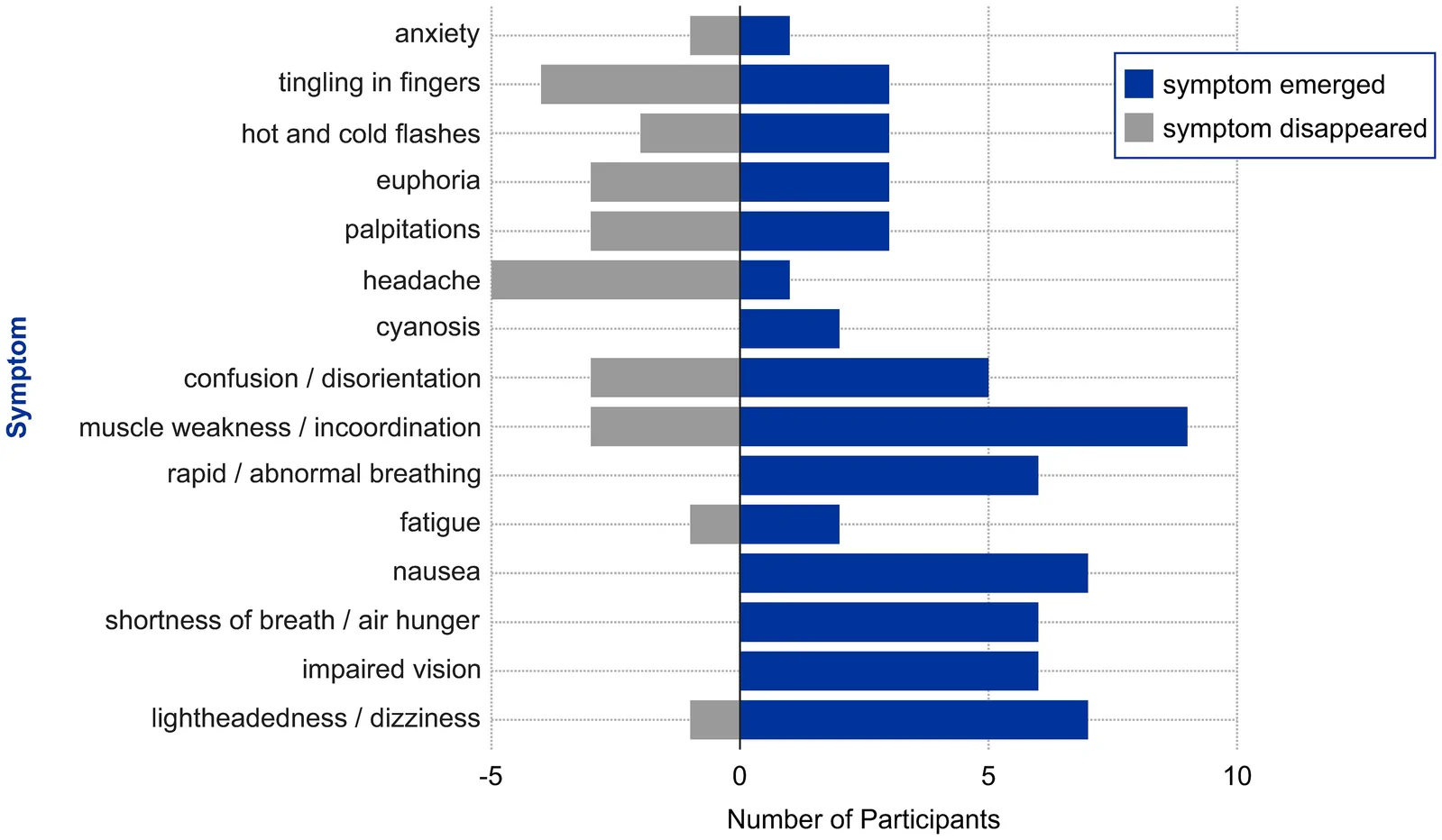
Changes in the occurrence of subjective symptoms between the control and hypoxia conditions. Blue bars indicate the number of participants who did not report a given symptom in the control condition but reported it under hypoxia (symptom emerged), while grey bars indicate the number of participants who reported the symptom in the control condition but not under hypoxia (symptom disappeared). Positive values represent symptom emergence, and negative values represent symptom disappearance.

**Figure 3** provides a descriptive overview of changes in symptom occurrence when transitioning from the control to the hypoxia condition. Several symptoms, including lightheadedness/dizziness, impaired vision, shortness of breath/air hunger, nausea, fatigue, and muscle weakness/incoordination, more frequently emerged under hypoxia, indicating an increased likelihood of symptom occurrence under hypoxia. In contrast, a smaller number of symptoms, such as headache and tingling in fingers, showed a tendency to disappear rather than emerge.

### 3.2 Continuous subjective evaluation

Analysis of the AUC revealed significantly lower ratings under hypoxia compared to the control condition for all three questions. Overall feeling, concentration, and breathing showed significant condition-related differences after FDR correction, accompanied by large effect sizes, see **Table 3**.

**Table 3:** Comparison of subjective ratings between control and hypoxia conditions.

AUC					
Question	Median diff.	Non-zero pairs	*r*_*rb*_	95% CI	*P*FDR
Overall feeling	−13	23	−0.78	[−0.97, −0.46]	0.0017
Concentration	−7	22	−0.60	[−0.93, −0.19]	0.013
Breathing	−16	23	−0.87	[−1.00, −0.61]	*<* 0.001
Mean					
Question	Median diff.	Non-zero pairs	*r*_*rb*_	95% CI	*P*FDR
Overall feeling	−1.00	23	−0.79	[−0.98, −0.51]	0.0014
Concentration	−0.57	21	−0.65	[−0.97, −0.25]	0.0095
Breathing	−1.29	23	−0.87	[−1.00, −0.58]	*<* 0.001
Slope					
Question	Median diff.	Non-zero pairs	*r*_*rb*_	95% CI	*P*FDR
Overall feeling	−0.09	23	−0.42	[−0.78, 0.04]	0.116
Concentration	≈ 0	23	−0.11	[−0.56, 0.38]	0.648
Breathing	−0.11	23	−0.69	[−0.93, −0.33]	0.011

Median diff. denotes the median of within-subject differences (hypoxia minus control). Non-zero pairs denote the number of paired observations with a non-zero difference. *r_rb_* denotes the matched-pairs rank-biserial correlation, with positive values indicating higher ratings under hypoxia and negative values indicating lower ratings under hypoxia. 95% CIs denote percentile bootstrap confidence intervals. *p*_FDR_ indicates FDR-adjusted *p*-values.

Results are presented for area under the curve (AUC), mean ratings across the exposure period (Mean), and temporal trends quantified by slope (Slope). Statistical comparisons were performed using paired Wilcoxon signed-rank tests with false discovery rate (FDR) correction.

Mean-based analyses yielded results consistent with the AUC findings, with significantly lower ratings for overall feeling, concentration, and breathing under hypoxia compared to the control condition, see **Table 3**. As all participants completed the same exposure duration, the AUC and mean values are mathematically related and are presented as complementary descriptors of the same underlying data rather than as independent outcome measures.

The analysis of temporal trends revealed a significant condition-related difference only for subjective breathing ratings, see **Table 3**. The slope of breathing ratings over time was significantly steeper under hypoxia than under the control condition, indicating a progressively worsening perception of breathing during hypoxic exposure. No significant time-related differences were observed for overall feeling or concentration. This effect was accompanied by a large effect size, reflecting a consistent temporal pattern across participants.

**Figure 4:**
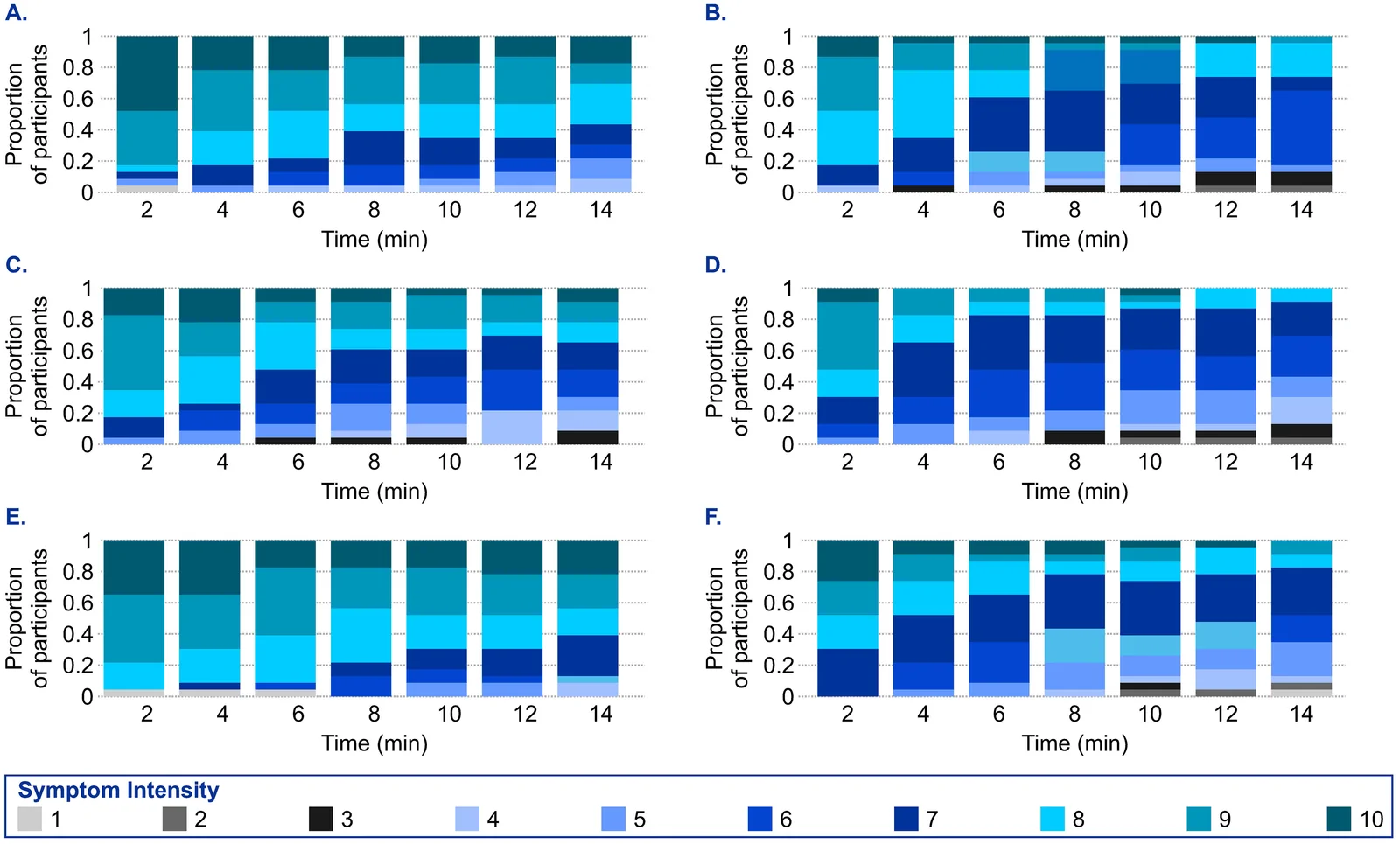
Reported intensities of the continuous subjective assessment over time. Stacked bars show the full distribution of symptom intensity ratings at each time point. Distributions of ratings for overall feeling **(A, B)**, concentration **(C, D)**, and breathing **(E, F)** are shown for the control **(A, C, E)** and hypoxia **(B, D, F)** conditions. Ratings were provided on a discrete intensity scale from 1 to 10. Bars represent the proportion of participants reporting each intensity level at a given time point.

Notably, while overall feeling and concentration exhibited a global downward shift under hypoxia, only breathing demonstrated a pronounced temporal deterioration, as indicated by the slope analysis. This pattern is also evident in the distribution of reported intensities over time shown in **Figure 4**.

### 3.3 Objective data

**Figure 5:**
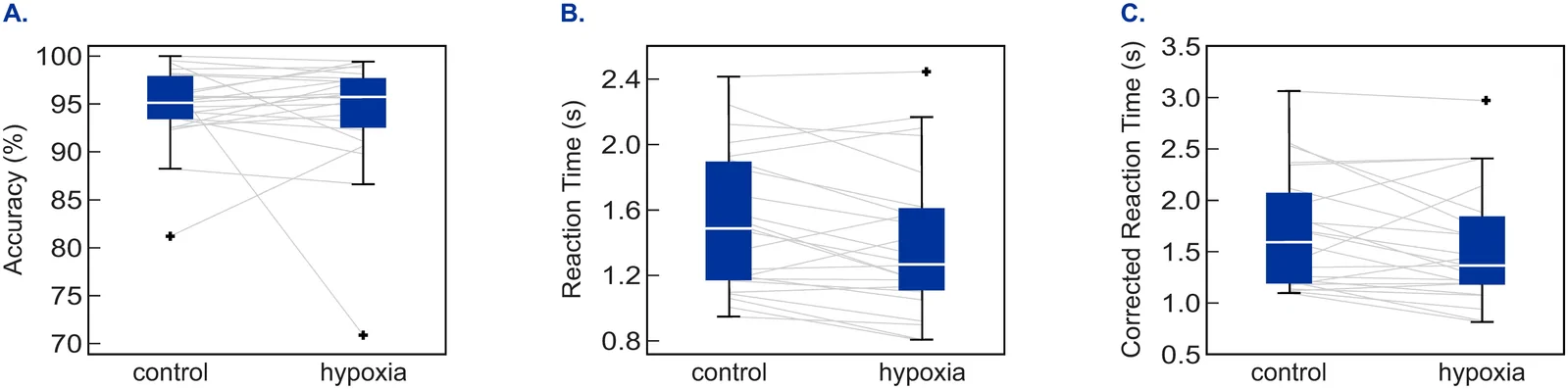
Distributions of Stroop task performance metrics in the form of boxplots showing accuracy **(A)**, mean reaction time of correct responses **(B)**, and accuracy-corrected reaction time **(C)** under control and hypoxia conditions. Grey lines represent paired participant data; black crosses indicate outliers.

**Figure 6:**
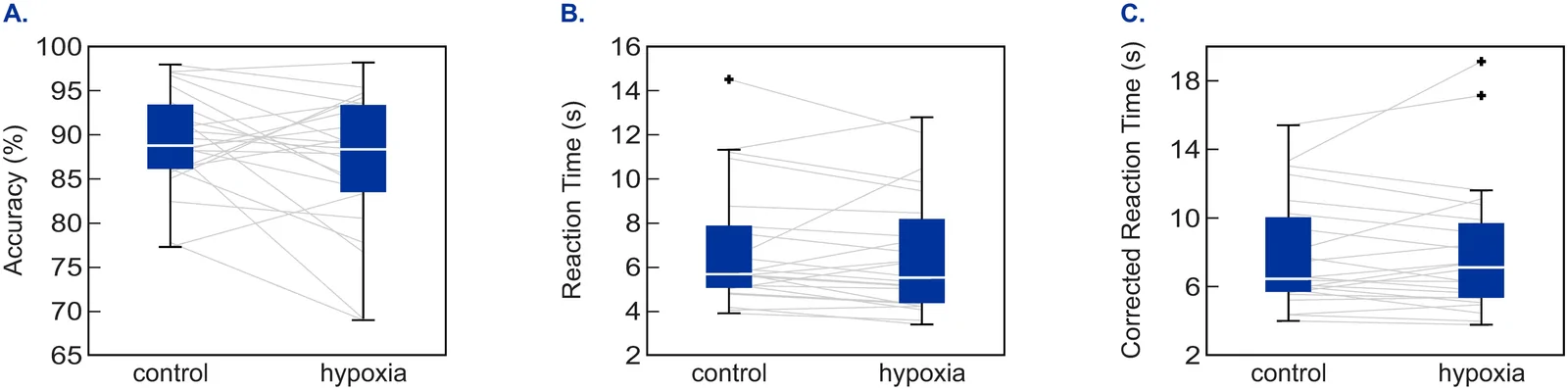
Distributions of mental rotation task performance metrics in the form of boxplots showing accuracy **(A)**, mean reaction time of correct responses **(B)**, and accuracy-corrected reaction time **(C)** under control and hypoxia conditions. Grey lines represent paired participant data; black crosses indicate outliers.

When examining objective performance measures, no statistically significant effects of hypoxia were observed for task accuracy in either the Stroop or mental rotation tasks (**Table 4**). In the ST, reaction times for correct responses and accuracy-corrected reaction times showed trends towards faster responses under hypoxia (**Figure 5**), accompanied by moderate effect sizes; however, these effects did not remain significant after FDR correction. Accuracy-corrected reaction times did not reveal additional effects beyond those observed for reaction times of correct responses. No significant effects or consistent trends were observed for any performance metric in the mental rotation task (**Figure 6**).

**Table 4:** Comparison of objective performance measures between control and hypoxia conditions for the Stroop test and mental rotation task.

Stroop test					
Outcome	Med. diff.	Non-zero pairs	*r*_*rb*_	95% CI	*P*FDR
Accuracy (%)	0.08	23	0.08	[−0.40, 0.54]	0.738
Mean RT (correct only)	−0.15	23	−0.54	[−0.87, −0.11]	0.073
RTcorr	−0.14	23	−0.46	[−0.83, −0.02]	0.077
Mental rotation					
Outcome	Med. diff.	Non-zero pairs	*r*_*rb*_	95% CI	*P*FDR
Accuracy (%)	−1.91	23	−0.34	[−0.74, 0.13]	0.229
Mean RT (correct only)	−0.51	23	−0.34	[−0.81, 0.13]	0.229
RTcorr	−0.23	23	−0.06	[−0.54, 0.41]	0.808

Median diff. denotes the median of within-subject differences (hypoxia minus control). Non-zero pairs denote the number of paired observations with a non-zero difference. *r_rb_*denotes the matched-pairs rank-biserial correlation, with positive values indicating higher values under hypoxia and negative values indicating lower values under hypoxia. 95% CIs denote percentile bootstrap confidence intervals. RT_corr_ represents accuracy-corrected reaction time computed from all responses. *p*_FDR_ indicates FDR-adjusted *p*-values.

Results are based on paired Wilcoxon signed-rank tests with false discovery rate (FDR) correction.

Physiological data confirmed a robust effect of hypoxic exposure on peripheral oxygen saturation. Mean SpO_2_, minimum SpO_2_, AUC, and the slope of SpO_2_ were all significantly lower under hypoxia than under the control condition (all FDR-adjusted *p <* 0.001; **Table 5**).

**Table 5:** Comparison of physiological oxygen saturation (SpO_2_) metrics between control and hypoxia conditions.

Metric	Median diff.	Non-zero pairs	*r*_*rb*_	95% CI	*P*FDR
Mean SpO_2_	−17.63	23	−1.00	[−1.00, −1.00]	2.7 × 10^−5^
Minimum SpO_2_	−24.00	23	−1.00	[−1.00, −1.00]	2.7 × 10^−5^
AUC SpO_2_	−271.00	23	−1.00	[−1.00, −1.00]	2.7 × 10^−5^
Slope SpO_2_	−1.14	23	−1.00	[−1.00, −1.00]	2.7 × 10^−5^

Median diff. denotes the median of within-subject differences (hypoxia minus control). Non-zero pairs denote the number of paired observations with a non-zero difference. *r_rb_*denotes the matched-pairs rank-biserial correlation, with negative values indicating lower SpO_2_ values under hypoxia. 95% CIs denote percentile bootstrap confidence intervals. *p*_FDR_ indicates FDR-adjusted *p*-values. Comparisons were performed using paired Wilcoxon signed-rank tests with false discovery rate (FDR) correction.

**Figure 7:**
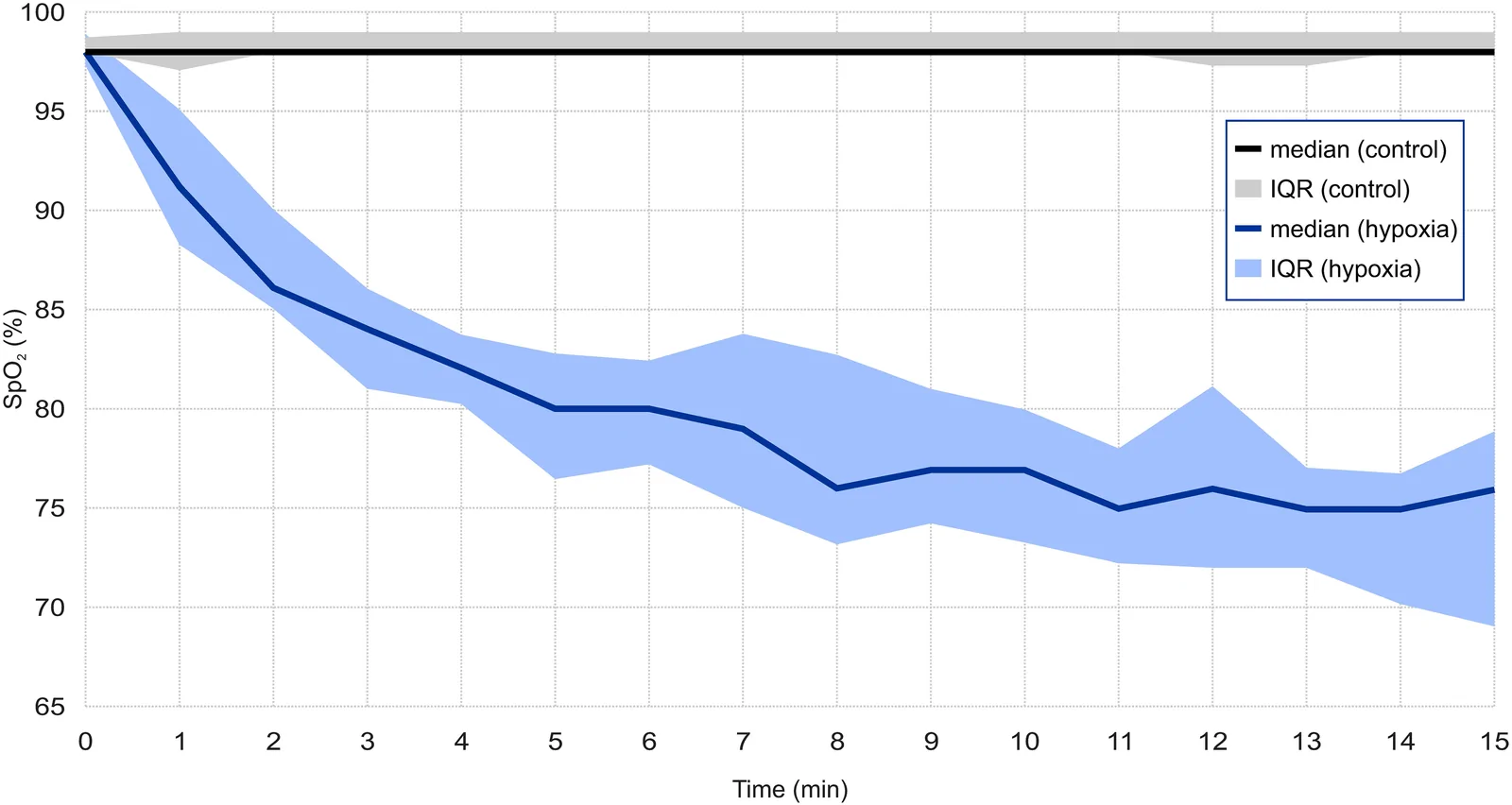
SpO_2_ levels over time during the control and hypoxia conditions, shown as medians with interquartile ranges (IQR). Measurements were obtained once per minute from 0 to 15 minutes.

Effect sizes were maximal for all SpO_2_ metrics (matched-pairs rank-biserial correlation *r_rb_*= −1.00), indicating a consistent decrease in oxygen saturation during hypoxia across all participants (**Table 5**). In addition to a global downward shift, hypoxia was characterized by a pronounced temporal decline in SpO_2_, as reflected by significantly steeper negative slopes over time. Differences in oxygen saturation between the control and hypoxia conditions are further illustrated in **Figure 7**.

Associations between physiological oxygen saturation metrics and subjective breathing ratings were explored using Spearman correlation analysis. No significant associations were detected between SpO_2_ metrics and subjective breathing ratings (**Table 6**). Neither cumulative hypoxic load (AUC SpO_2_) nor the temporal decline in oxygen saturation (SpO_2_ slope) was significantly related to the corresponding subjective breathing metrics (all FDR-adjusted *p* = 0.61). Minimum SpO_2_ was also not significantly associated with mean subjective breathing ratings.

**Table 6:** Associations between oxygen saturation (SpO_2_) metrics and subjective breathing ratings under hypoxia.

SpO_2_ metric	Subjective metric	Spearman *ρ*	95% CI	*P*FDR
AUC SpO_2_	Breathing AUC	−0.11	[−0.54, 0.36]	0.61
Slope SpO_2_	Breathing slope	0.18	[−0.23, 0.55]	0.61
Minimum SpO_2_	Mean breathing	−0.23	[−0.60, 0.23]	0.61

Correlations were computed for the hypoxia condition only. 95% CIs denote percentile bootstrap confidence intervals. *p*_FDR_ denotes FDR-adjusted *p*-values. Spearman correlation coefficients with 95% bootstrap confidence intervals and false discovery rate (FDR)-adjusted *p*-values are reported.

**Figure 8:**
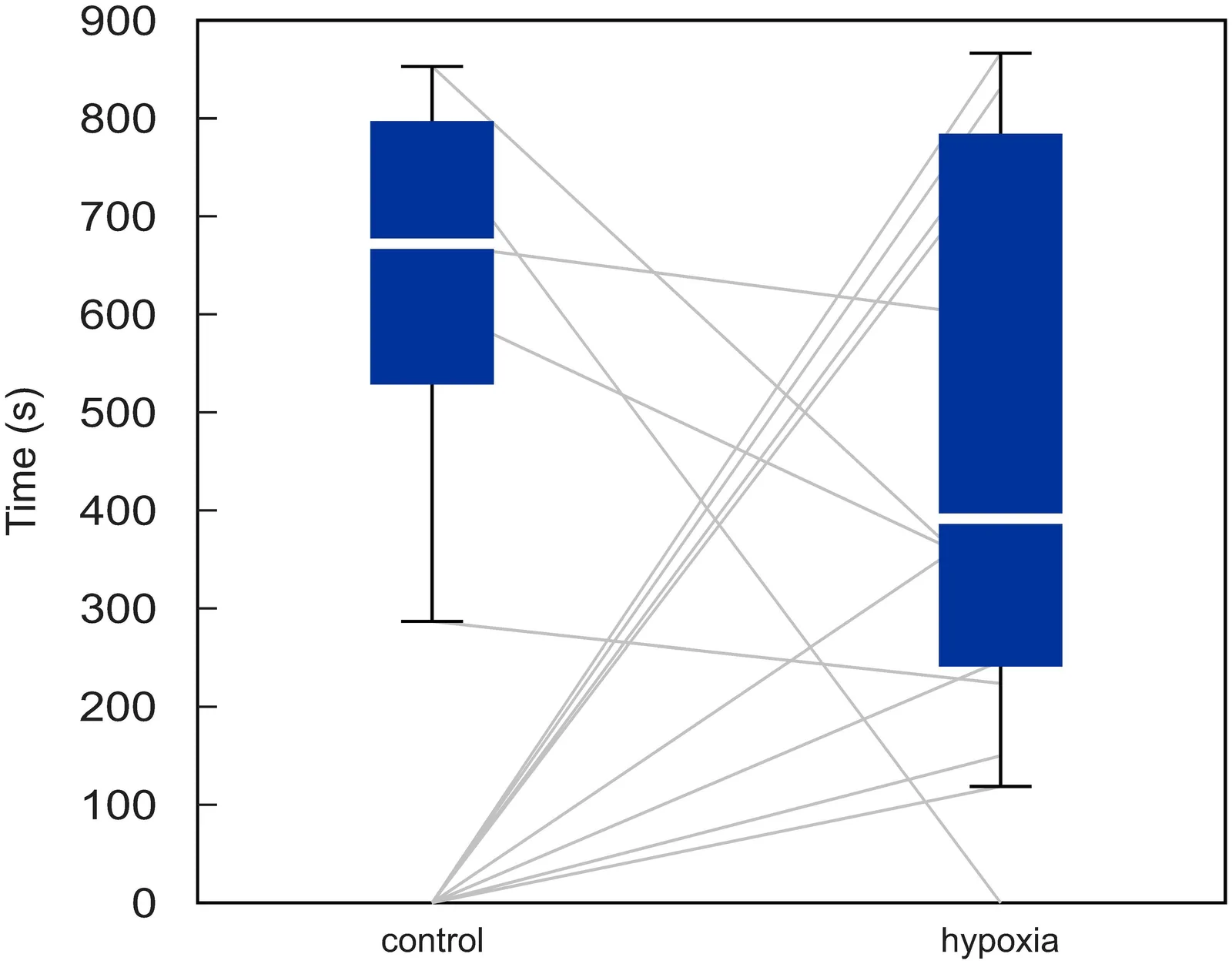
Distributions of the time to hypoxia recognition shown as boxplots for the control and hypoxia conditions. Boxplots include only participants who reported hypoxia recognition (time *>* 0 s). Participants who did not report hypoxia recognition were assigned a value of 0 s solely for visualization of the paired observations; no participant reported hypoxia recognition at time = 0 s, and these values were not included in the boxplot statistics. Light grey lines show paired observations for all participants.

Regarding individual recognition of hypoxia, participants were asked to explicitly report the moment at which they became aware of entering a hypoxic state during each experimental session. During the hypoxia condition, participants reported hypoxia significantly more often than during the control condition (exact McNemar test, *p* = 0.0215). The observed change was driven primarily by transitions from non-reporting to reporting hypoxia (0→1; *n* = 9), with substantially fewer transitions in the opposite direction (1→0; *n* = 1; **Figure 8**). Overall, the prevalence of hypoxia recognition increased from 21.7% in the control condition to 56.5% under hypoxia, corresponding to an absolute increase of 34.8 percentage points.

## 4 Discussion

The physiological data confirmed that the hypoxic manipulation was robust and consistent across all participants. All SpO_2_ metrics showed large and uniform effects, including lower mean and minimum values, reduced cumulative oxygen saturation (AUC), and a pronounced temporal decline during hypoxia. This confirms that all participants were exposed to a comparable hypoxic load and that subsequent subjective and performance-related findings can be interpreted in the context of a reliably induced physiological stressor.

Although several symptoms commonly associated with hypoxia exhibited consistent directional changes, none remained statistically significant after correction for multiple comparisons. This outcome likely reflects substantial inter-individual variability in subjective symptom perception under hypoxic exposure combined with the conservative nature of the FDR correction. Nevertheless, several symptoms were at the borderline of statistical significance, including lightheadedness/dizziness, impaired vision, air hunger, and nausea, which correspond to well-established early hypoxia symptoms and have been reported in previous studies (Woodrow et al., 2011; Chiang et al., 2021b).

Analyses of symptom occurrence further support this interpretation. Although changes in symptom prevalence between conditions did not reach statistical significance after correction, several symptoms emerged predominantly under hypoxia or were reported more frequently compared to the control condition. These findings indicate consistent trends toward increased symptom occurrence during hypoxic exposure; however, they should be interpreted as descriptive rather than confirmatory.

Importantly, the increased prevalence of some symptoms under hypoxia was driven primarily by reports of mild intensity, particularly in the case of impaired vision. This suggests that hypoxia increased the likelihood of symptom occurrence rather than inducing uniformly severe symptoms across participants. This pattern is consistent with early or moderate hypoxic exposure, in which subtle perceptual and sensory changes may precede more pronounced symptom severity (Davis et al., 2008). Comparable tendencies were also observed for other symptoms, such as rapid or abnormal breathing and muscle weakness or incoordination. Given that the hypoxic exposure in the present study was time-limited and conducted at a moderate simulated altitude, it is plausible that prolonged or more severe hypoxia would result in stronger symptom intensities.

The increase in breathing-related symptoms is further supported by the continuous subjective evaluation. This observation is physiologically plausible, as hypoxic stimulation of peripheral chemoreceptors increases ventilatory drive (Prabhakar and Peng, 2004), which may contribute to the subjective perception of respiratory discomfort even in the absence of objectively measured respiratory parameters. However, objective respiratory parameters such as breathing frequency or minute ventilation were not measured in the present study. Therefore, the observed breathing discomfort should be interpreted as a subjective perception that may have been influenced by ventilatory responses rather than as direct evidence of hyperventilation.

While overall feeling and concentration showed a general downward shift under hypoxia, only subjective breathing ratings demonstrated a significant temporal deterioration. The selective emergence of a temporal effect for breathing, but not for overall feeling or concentration, suggests that respiratory discomfort was the only subjective measure to demonstrate a significant temporal change in the present study and may therefore represent a particularly responsive indicator of hypoxic exposure under the present experimental conditions. However, this interpretation should be considered in the context of the fixed-order experimental design. In contrast, more global or cognitively framed self-assessments appear to be less sensitive to gradual hypoxic changes and exhibit greater inter-individual variability.

At the same time, no significant association was detected between subjective breathing ratings and physiological SpO_2_ metrics. This finding indicates that, within the present dataset, perceived breathing discomfort was not directly associated with the degree of oxygen desaturation. However, this absence of a detectable association should be interpreted with caution, as it may reflect the limited sample size in addition to the considerable inter-individual variability in hypoxia perception (Davis et al., 2008). Subjective awareness of hypoxia may therefore be influenced not only by the degree of oxygen desaturation but also by individual sensitivity, expectancy, and perceptual factors.

Although the cognitive tasks were included primarily to increase mental workload during hypoxic exposure and their analysis should therefore be regarded as exploratory, no measurable impairment of cognitive performance was detected in the Stroop and mental rotation tasks under the present experimental conditions. This finding contrasts with results reported by Bouak et al. (2018), who observed performance decrements under hypoxic exposure; however, the experimental conditions are not directly comparable in terms of simulated altitude, exposure duration, and overall study design. In contrast, other studies have reported no significant effects of hypoxia on cognitive performance under different experimental conditions (Oniscenko et al., 2024). Legg et al. (2016) similarly found no significant impairment in complex cognition, including Stroop performance, spatial reasoning, and mathematical processing, at simulated altitudes of 8000 and 12000 ft, despite measurable physiological effects of hypoxia. Steinman et al. (2023) likewise observed that response accuracy in a visual choice reaction task was maintained under hypoxia at 15000 ft in experienced military pilots, even when reaction time was significantly prolonged. Notably, both of these studies employed considerably milder hypoxic exposures than the present study, which used a 9% oxygen mixture corresponding to a simulated altitude of 20300 ft. Taken together, these exploratory findings indicate that no measurable impairment was detected using the selected accuracy-based cognitive measures under the present experimental conditions. However, subtle cognitive effects cannot be excluded, and the observed pattern should be interpreted in the context of the fixed-order study design and possible practice effects.

Despite clear physiological evidence of hypoxia, not all participants reported conscious awareness of entering a hypoxic state, which is consistent with previous findings (Leinonen et al., 2020). Nevertheless, hypoxia recognition occurred significantly more often during the hypoxia condition than during the control condition, indicating that subjective awareness of hypoxia is neither universal nor immediate. This highlights substantial inter-individual variability in hypoxia perception and supports the relevance of targeted hypoxia awareness training, which has been shown to improve recognition performance (Leinonen et al., 2020; Johnston et al., 2012; Tu et al., 2020; Chiang et al., 2021b). This concept is also reflected in aviation medicine through the time of useful consciousness (TUC), which is typically presented as an average estimate rather than a fixed threshold and is known to exhibit considerable inter-individual variability (Federal Aviation Administration, 2015). Furthermore, published TUC values are primarily derived from hypobaric chamber studies and should not be directly extrapolated to normobaric hypoxia induced using a ROBD. Together, these considerations further emphasize that individual recognition of hypoxia, rather than reliance on average physiological expectations, remains a fundamental objective of hypoxia awareness training.

This is consistent with current aviation practice, where practical hypoxia awareness training constitutes a standard component of military flight training and has repeatedly been shown to improve subsequent recognition of individual hypoxia symptoms during follow-up hypoxic events (Chiang et al., 2021b; Tristan, 2017; Singh et al., 2010; Smith, 2008; Leinonen et al., 2020). In contrast, hypoxia training in civil aviation is largely limited to theoretical instruction, despite surveys indicating that many civilian pilots consider the current training insufficient (Patrao et al., 2013). Furthermore, repeated hypoxia awareness training has been shown to reinforce recognition of individual symptom profiles, with pilots consistently reporting similar symptoms across repeated exposures (Smith, 2008; Johnston et al., 2012; Tu et al., 2020).

Although hypobaric chambers remain the traditional approach for hypoxia awareness training, normobaric hypoxia induced using a reduced oxygen breathing device (ROBD) has been increasingly adopted as an alternative. Previous studies suggest that normobaric hypoxia elicits subjective symptoms comparable to those observed during hypobaric exposure, is suitable for hypoxia awareness training (Singh et al., 2010; Artino et al., 2006; Kumar et al., 2013), and may produce comparable physiological responses under controlled experimental conditions (Singh et al., 2010). Nevertheless, direct physiological equivalence between normobaric and hypobaric hypoxia has not been fully established, and further comparative studies are warranted before findings can be directly extrapolated between these modalities. Therefore, the findings of the present study should be interpreted specifically in the context of normobaric hypoxia induced using ROBD.

Several observations suggest that expectation effects may have contributed to subjective responses. Fourteen out of fifteen symptoms were reported by at least some participants during the control condition, and 21.7% of participants reported hypoxia recognition despite the absence of physiological hypoxia. Together, these findings suggest that expectation effects and the non-specific nature of several hypoxiarelated symptoms may have influenced symptom reporting and hypoxia recognition. In addition, certain symptoms may have been influenced by the experimental setup itself; for example, tingling in the fingers may have been related to the use of the pulse oximeter rather than hypoxia per se. Finally, because symptom presence was assessed only after each experimental session without a pre-exposure baseline, it was not possible to distinguish symptoms that developed during the experiment from those already present before exposure.

These findings should also be discussed in the context of the experimental design. Unlike large military studies that rely primarily on retrospective symptom reports collected during hypobaric chamber training (e.g. Woodrow et al. (2011)), the present study employed a within-subject comparison of two consecutive experimental runs. This design requires participants to actively monitor their internal state, which may increase self-awareness, expectancy effects, or stress-related responses. As a result, hypoxia recognition may partially resemble a detection task rather than a purely spontaneous perceptual experience, potentially contributing to symptom reporting in both conditions. At the same time, the paired design allows for a direct statistical comparison between conditions and enables symptom patterns to be evaluated relative to a well-defined physiological manipulation.

An important limitation of the present study is the fixed session order, with the control condition always preceding hypoxia. Although a randomized or counterbalanced design would reduce potential order and practice effects, it would also introduce other methodological challenges. In particular, prior hypoxia exposure could influence symptom awareness and subjective reporting during a subsequent normoxic session, while a longer washout period and a substantially prolonged experimental protocol would likely be required to minimize potential carry-over effects. The selected design therefore represents a methodological trade-off between minimizing carry-over effects and minimizing order-related confounding. Consequently, some influence of session order, including learning effects, increased task familiarity, expectancy, and heightened attention to internal sensations during the second session, cannot be excluded when interpreting the present findings. Although each experimental session lasted only 15 minutes and was separated by a recovery period, making substantial fatigue-related effects less likely, some contribution of fatigue cannot be entirely excluded.

Additional sources of variability may be attributed to technical aspects of the experimental setup. Although the custom-developed software enabled precise timing, synchronized task delivery, and structured data collection, minor technical limitations were present. For instance, the Czech translation of the Stroop task may have altered task difficulty due to linguistic similarities between color names. While these factors are unlikely to have substantially influenced the primary outcomes, they represent sources of uncontrolled variance that should be addressed in future studies. However, these measures were not primary study outcomes, and the cognitive tasks were included primarily to increase mental workload during the experimental sessions.

An additional limitation relates to the pulse oximetry measurements. A commercially available fingertip pulse oximeter was used primarily to verify hypoxic exposure and ensure participant safety rather than for high-resolution physiological monitoring. Although the device provides a manufacturer-specified accuracy of ±2% within the 70–100% SpO_2_ range, its accuracy below this range is not specified. Furthermore, pulse oximetry measurements are known to be affected by factors such as motion artifacts, peripheral perfusion, and skin pigmentation (León-Valladares et al., 2024). In addition, SpO_2_ was recorded once per minute, which does not allow detailed characterization of the temporal dynamics of oxygen desaturation and may fail to capture short-term fluctuations in oxygen saturation. However, the primary purpose of these measurements was to confirm successful induction of hypoxia and ensure participant safety rather than to characterize the fine temporal profile of SpO_2_ changes. Given the standardized experimental conditions, with gradual exposure to a fixed hypoxic gas mixture and no additional physiological stressors such as physical activity, the selected sampling interval was considered sufficient for this purpose. These limitations should be considered when interpreting the absolute SpO_2_ values. Nevertheless, they are unlikely to affect the principal conclusion that a marked and consistent oxygen desaturation occurred during hypoxia.

It should also be noted that the absence of statistically significant effects for several outcomes is likely related to the relatively small sample size (*n* = 23) and the application of conservative FDR correction. The sample size was primarily determined by the practical constraints associated with the controlled hypoxia protocol. Given the modest sample size, sparse symptom ratings, and correction for multiple comparisons, the study had limited sensitivity to detect small-to-moderate or inconsistent withinsubject effects. Consequently, non-significant findings should be interpreted cautiously and should not be considered evidence of the absence of an effect. The study population was also relatively young and comprised both active pilots and non-pilots selected according to predefined aviation-relevant medical criteria. Although this approach was intended to reflect a fit-to-fly population, these characteristics may limit the generalizability of the findings to the broader operational aircrew population, particularly older or more operationally experienced flight crews. Furthermore, the controlled laboratory setting and participants’ awareness of potential hypoxic exposure may have influenced subjective reporting.

Overall, while the results should be interpreted with appropriate caution, they provide further insight into the complex relationship between physiological hypoxia and subjective experience. The findings suggest that hypoxia primarily affects physiological state and subjective perception, while breathing-related sensations demonstrated the clearest temporal changes among the subjective measures and may have contributed to participants’ awareness of hypoxia under the present experimental conditions.

From an operational perspective, these findings further support the importance of practical hypoxia awareness training aimed at improving recognition of individual symptom profiles. While such training is routinely implemented in military aviation, the present results may also contribute to ongoing discussions regarding the role of practical hypoxia awareness training in civilian pilot education.

## 5 Conclusion

This study investigated subjective symptom perception during experimentally induced hypoxia, with a primary focus on post-exposure symptom reporting, continuous self-assessment, and individual recognition of hypoxic state. Although none of the individual subjective symptoms reached statistical significance after correction for multiple comparisons, several symptoms showed consistent directional changes and approached borderline statistical significance after correction for multiple comparisons. Together with the continuous subjective ratings, these findings suggest that hypoxic exposure influenced subjective perception, although the magnitude of these effects should be interpreted in light of the study design and sample size. Continuous self-reporting revealed that breathing-related sensations demonstrated the clearest temporal changes among the subjective measures in the present study, and more than half of the participants recognized that they were experiencing hypoxia during the hypoxia condition.

Several limitations should be acknowledged. The relatively small sample size, together with the conservative FDR correction, may have limited the ability to detect small-to-moderate or inconsistent effects. In addition, the fixed order of experimental conditions represents an important methodological limitation. Although this approach was intended to minimize potential carry-over effects of prior hypoxia exposure on subsequent normoxic testing, it did not eliminate the possibility of order-related confounding, including increased task familiarity, expectancy, and heightened attention to internal sensations during the second experimental session. Consequently, some influence of session order on both subjective and objective outcomes cannot be excluded. Finally, the experiment was conducted under controlled laboratory conditions, which may have influenced subjective reporting through expectancy or increased self-monitoring. Furthermore, the controlled laboratory setting may limit the generalizability of the findings to operational aviation environments.

Nevertheless, the physiological data confirmed that hypoxia was robustly and consistently induced across all participants, as evidenced by pronounced reductions in oxygen saturation and clear temporal declines in SpO_2_. In this context, the observed subjective responses provide valuable insight into how hypoxia is perceived. Breathing-related sensations demonstrated the clearest temporal changes among the subjective measures; however, their lack of a direct association with physiological SpO_2_ metrics suggests that subjective hypoxia awareness reflects more than the degree of oxygen desaturation alone. Together, these findings highlight the complex and individually variable relationship between physiological hypoxia, subjective experience, and cognitive performance. Although no measurable impairment was detected using the selected cognitive tasks, subtle cognitive effects cannot be excluded.

Future studies should aim to increase sample size and explore a wider range of hypoxic exposures, including longer durations or more severe hypoxia, to further clarify the relationship between physiological load, subjective perception, and performance. Involving larger cohorts and more complex experimental protocols may enable the implementation of randomized or counterbalanced designs, allowing the effects of hypoxia to be disentangled more effectively from potential order-related influences while maintaining adequate control of carry-over effects. Alternative or more sensitive performance measures may help capture subtle cognitive effects that were not evident in the present design. Overall, the findings support the relevance of subjective symptom monitoring and hypoxia awareness training, while emphasizing the need for carefully designed experimental approaches to better understand individual differences in hypoxia recognition.

## Data Availability

The raw data supporting the conclusions of this article will be made available by the authors, without undue reservation.
